# RANKL-induced M1 macrophages are involved in bone formation

**DOI:** 10.1038/boneres.2017.19

**Published:** 2017-10-17

**Authors:** Rong Huang, Xin Wang, Yinghong Zhou, Yin Xiao

**Affiliations:** 1 The Institute of Health and Biomedical Innovation, Queensland University of Technology, Brisbane, Queensland, Australia; 2 Department of Orthopaedic Surgery, Affiliated Hospital of Zunyi Medical University, Zunyi, Guizhou, China

## Abstract

The activation of M1 macrophages can be achieved by stimulating them with lipopolysaccharide (LPS) and interferon-γ (IFN-γ). However, M1 can be found under physiological conditions without any pathological stimuli. This study aimed to understand the involvement of RANKL-induced M1 macrophages in bone formation compared with pathologically induced macrophages. Fischer rats were used to investigate macrophage distribution in normal and injured femoral condyles *in vivo*. Bone marrow-derived macrophages (BMDMs) were activated with LPS+IFN-γ and RANKL to achieve M1 activation *in vitro*. Gene expression related to inflammation, osteoclastogenesis, angiogenesis, and migration was determined by reverse transcription-quantitative polymerase chain reaction (RT-qPCR) and fluorescence-activated cell sorting (FACS). Tissue macrophages showed distinct expression patterns at different bone regions. RANKL was found in close proximity to inducible nitric oxide synthase-positive (iNOS+) cells *in vivo*, suggesting an association between RANKL expression and iNOS+ cells, especially in trabecular bone. RANKL-induced macrophages showed a different cytokine secretion profile compared with pathologically induced macrophages. Both osteoclasts and M1 macrophages peaked on day 7 during bone healing. RANKL could trigger M1-like macrophages with properties that were different from those of LPS+IFN-γ-induced macrophages. These RANKL-activated M1 macrophages were actively involved in bone formation.

## Introduction

Macrophages are a heterogeneous population of hematopoietic origin that are involved in crucial innate immune defense and have tissue-specific functions in the regulation and maintenance of organ homeostasis. Tissue macrophages are identified in many tissues and have two key functions: (1) to respond to pathogens and modulate the adaptive immune responses and (2) to facilitate tissue repair and regeneration.^1,2^ As heterogeneous cells with great plasticity, macrophages activated by interferon-γ (IFN-γ), and lipopolysaccharide (LPS) achieve a “classically activated” or a “killer” phenotype (CAM, M1) characterized by high level secretion of pro-inflammatory cytokines and the production of reactive nitrogen and oxygen intermediates. The current viewpoint on this process emerged from the inflammation model of immune responses and was linked with type 1 T helper cell responses and IFN-γ production by antigen-activated immune cells.^3^ When exposed to cytokines secreted by type 2 T helper cells or other glucocorticoids and secosteroids, an alternative activated phenotype (M2) can be achieved with a profile that includes a strong expression of mannose receptors, scavenging molecules, and a high level production of ornithine and polyamines, which participate in parasite encapsulation, angiogenic promotion, and matrix remodeling.^4,5^ The concept of multiple macrophage activation states is not new. However, extending this idea to tissue macrophages has gained increased interest in recent years because these unique phenotypes likely reflect the different functions and influence of the tissue environment in which they reside.^6^

Tissue macrophages are closely associated with common diseases. M1 macrophages have been reported to promote atherosclerosis by secreting pro-inflammatory cytokines.^7^ Although M2 macrophages help the blood vessels remove cholesterol, when the cholesterol level changes, the M2 macrophages become foam cells that contribute to the atheromatous plaques of atherosclerosis.^8^ In Crohn's disease, M2 macrophages, but not M1 macrophages, display lower tumor necrosis factor (TNF)-*α* levels, abnormal morphological maturation, prolonged intracellular bacterial survival, and different surface marker expression.^9^ In addition, an unrestrained pro-inflammatory M1 macrophage population induced by iron has been found to impair wound healing in humans and in mice. Conversely, M2 macrophages decrease the pro-inflammatory cytokine levels and secrete components of the extracellular matrix that may be essential in the late phases of tissue repair.^10^

The traditional notion of M1 macrophages has been formed under the influence of pathological stimuli such as IFN-γ and LPS. It is understandable because the innate immune system is usually activated by the induction of pathogens, vaccines, and tumors, and the innate immune response is often studied in the context of host defense. Macrophages are the critical effectors and regulators of inflammation, and their activation has been closely associated with pathogenic stimuli. Therefore, M1 macrophages are activated *in vitro* via the induction of LPS and IFN-γ to mimic the real environment. However, in some scenarios, such as in normal tissues, the traditional concept to investigate macrophage properties under inflammatory circumstances is no longer applicable. For example, M1 macrophages have been found in adipose tissues with the presence of integrin alpha X, where LPS and IFN-γ could not be applied.^11^ It is assumed that these non-pathologically activated cells may reflect the real nature of M1 macrophages.

RANKL as the receptor activator of nuclear factor-*κ*B ligand serves to activate osteoclasts, which are critically involved in bone resorption.^12,13^ RANKL triggers inducible nitric oxide synthase (iNOS) expression and nitric oxide (NO) release in a time- and dose-dependent manner that can be specifically blocked by the RANKL decoy receptor osteoprotegerin. The inhibition of RANKL-induced NO increases osteoclast formation and bone resorption, which suggests that RANKL-regulated osteoclastogenesis could be restrained by NO production.^14^ Because iNOS expression is elevated in M1 macrophages,^15^ it is of great interest to investigate whether M1 macrophages can be activated with the induction of RANKL and to compare the differences between RANKL- and LPS+IFN-γ-induced macrophages. Although the rough dichotomy between physiologically induced (RANKL) and pathologically induced (LPS+IFN-γ) macrophages does not fully capture the entire heterogeneity and complexity found among tissue macrophages, the goal of this study is to highlight the emerging interest in the unrestrained M1 macrophage and to provide a molecular basis for the development of novel approaches to prevent, manage, and treat bone-related diseases.

## Materials and methods

### Immunohistochemical and immunofluorescent staining

Six male Fischer rats (Animal Resources Centre, Canning Vale, Western Australia, Australia) at 6 weeks of age were used in the *in vivo* study, which was approved by the Animal Ethics Committee of Queensland University of Technology (Approval number: 1400000023). Formalin-fixed and paraffin-embedded cortical bone and trabecular bone tissues were cut into 5-μm-thick slices followed by paraffin removal in xylene and rehydration with a graded ethanol series (100%, 90%, and 70%) for 5 min each. The sections were boiled in tri-sodium citrate buffer (pH 6.0) at 94 °C for 10 min to achieve antigen retrieval and then cooled down to room temperature. Specimens were used for either immunohistochemical staining or immunofluorescent staining. For immunohistochemical staining, samples were incubated with 3% H_2_O_2_ for 20 min to quench endogenous peroxidase enzymes and 1% bovine serum albumin (Sigma-Aldrich, St. Louis, MO, USA) blocking buffer for 30 min to reduce nonspecific binding. Primary antibodies against cluster of differentiation 68 (CD68; 1:300, ab125212, Abcam, Cambridge, UK), iNOS (1:200, ab15323, Abcam), C-C chemokine receptor type 7 (CCR7; 1:50, NB100–712, Novus Biologicals, Littleton, CO, USA), Arginase1 (1:100, sc-20150, Santa Cruz), cluster of differentiation 163 (CD163; 1:100, MCA342GA, AbD Serotec, Raleigh, NC, USA), and isotype controls, including rabbit IgG (N1699, DAKO, Carpinteria, CA, USA), mouse IgG1 (N1698, DAKO), and goat IgG (ab37373, Abcam), were applied at optimal dilutions and incubated at 37 °C for 2 h. This process was followed by incubation with a corresponding anti-mouse/rabbit secondary antibody (K4061, DAKO) and an anti-goat secondary antibody (1:250, ab97110, Abcam) for 30 min at 37 °C. Diaminobenzidine (Dako) and Mayer's hematoxylin (Sigma-Aldrich) were used to visualize the staining. All stained sections were dehydrated through a series of graded alcohol baths of increasing concentration, cleared in xylene, and mounted with coverslips. Tissue macrophages that were identified by positive staining from three randomly selected sections were recorded, and the strength of the immunohistochemical staining was quantified. Color threshold and binary conversion were used, and the integrated density of the target area was measured using ImageJ (V1.50e Fiji, National Institutes of Health, Bethesda, MD, USA). For immunofluorescent staining, samples were permeabilized for 10 min with 0.1% Triton X-100 in phosphate-buffered saline (PBS) and blocked with 1% bovine bovine serum albumin for 30 min. Primary antibodies against iNOS (1:200, ab15323, Abcam) and RANKL (1:80, 12A668, Novus Biologicals) were applied at 37 °C for 2 h. Samples were then incubated with secondary antibodies including Alexa Fluor 488-conjugated anti-rabbit IgG (1:1000, #4412, Cell Signaling Technology, Danvers, MA, USA) and Alexa Fluor 568-conjugated anti-mouse IgG (1:1 000, A11004, Thermo Fisher Scientific) for 30 min at 37 °C. An Axio Imager M2 microscope (Zeiss) was used to observe the immunofluorescent intensity.

### Primary cell culture

Bone marrow-derived macrophages (BMDMs) were harvested from the bone marrow of six C57BL/6 mice (Animal Resources Centre). The isolation of bone marrow-derived cells was performed by density gradient centrifugation, and the red blood cells were removed using red blood cell lysing buffer (Sigma-Aldrich). After centrifugation, the pellet was dissociated and seeded in culture flasks supplemented with Gibco RPMI 1640 medium (Life Technologies, Carlsbad, CA, USA) containing 10% (v/v) fetal bovine serum and 1% (v/v) penicillin/streptomycin. After 4 h of incubation, the supernatant was collected and filtered through a cell filter with 70 μm pore size, and the adherent cells were kept for mesenchymal stromal cell (MSC) culture. The filtrate was then incubated in T75 flasks supplemented with 10 ng·mL^−1^ macrophage colony-stimulating factor (315-02, PeproTech, Rocky Hill, CT, USA). The same amount of fresh medium containing 10% fetal bovine serum, 1% penicillin/streptomycin, and 10 ng·mL^−1^ macrophage colony-stimulating factor was added to the same flask on the fourth day of cell culture. Cells were used for further analysis after 7-day culture.

### Macrophage activation

BMDMs were seeded in a six-well plate at an initial density of 5×10^5^ cells per well and induced with 100 ng·mL^−1^ LPS (L3012, Sigma-Aldrich), 100 ng·mL^−1^ IFN-γ (285-IF, R&D System), LPS+IFN-γ, and RANKL (462-TEC, R&D Systems, Minneapolis, MN, USA) at different concentrations (1, 10, and 100 ng·mL^−1^) for 12 h. Polarized cells were harvested for mRNA analysis by reverse reverse transcription-quantitative polymerase chain reaction (RT-qPCR).

### RT-qPCR

The total RNA of BMDMs induced with RANKL and LPS+IFN-γ for 12 h was extracted with TRIzol reagent (Ambion, Life Technologies) according to the manufacturer’s instruction. A DyNAmo cDNA Synthesis Kit (Thermo Fisher Scientific) was used for cDNA synthesis. An ABI PRISM 7500 FAST Sequence Detection System (Applied Biosystems) was used for RT-qPCR. The experiment was performed in triplicate, and the expression of the target genes was normalized to that of the endogenous control β-actin. The 2^−ΔΔCt^ method was used to analyze the relative expression levels.^16^ The corresponding primer sequences of the reference genes and the target genes are listed in Table 1.

### Flow cytometry analysis for macrophages

Cells including BMDMs and RANKL- or LPS+IFN-γ-primed BMDMs were harvested and washed twice with PBS. Pellets were resuspended in 300 μL PBS, and ice-cold 100% ethanol was added to achieve a total volume of 1 000 μL. The cells were left in 70% ethanol at 4 °C for 1 h. After centrifugation, ethanol was removed, and the cells were washed twice with PBS. The cells were then resuspended in 500 μL anti-iNOS solution (1:100, bs-2072R-A488, Bioss, Woburn, MA, USA) or anti-rabbit IgG solution (1:100, bs-0295P-A488, Bioss) for 30 min on ice in the dark. After the second wash, the cells were resuspended in 300 μL PBS and transferred to flow tubes. Flow cytometric analysis was performed using the FACSAria III Cell Sorter (BD Biosciences, San Jose, CA, USA).

### Macrophage-conditioned medium and co-culture with MSCs

BMDMs were either left untreated or treated with RANKL (for M1 polarization) and interleukin (IL)-4 (for M2 polarization) for 12 h. The cells were washed three times with PBS and incubated with serum-free Dulbecco's Modified Eagle Medium (Thermo Fisher Scientific) at 37 °C for another 12 h. The conditioned medium (CM) was collected and subjected to centrifugation at a speed of 1 000 *g* for 5 min. The supernatant was then aliquoted and stored at −80 °C. A standard osteogenic differentiation medium composed of Dulbecco's Modified Eagle Medium with 10% fetal bovine serum, 1% penicillin/streptomycin, 10 nmol·L^–1^ Dexamethasone, 50 μg·mL^−1^ ascorbic acid (Sigma-Aldrich), and 10 mmol·L^–1^ β-glycerophosphate (Sigma-Aldrich) was mixed with the CM at a 1:1 ratio. MSCs were cultured in this pre-mixed medium for up to 7 days, and the medium was changed every 2 days.

### Mineralization detection in co-culture samples

Mineralized nodules were visualized by Alizarin red S staining on day 7 of co-culture (MSCs cultured in pre-mixed CM). Samples were fixed in 4% paraformaldehyde (Life Technologies) for 10 min at room temperature. After rinsing with distilled water, samples were stained in 1% Alizarin red S (Sigma-Aldrich) for 20 min and then air-dried. Images were captured using the Zeiss Axiovision software. The samples were extracted using 300 μL of a 50% acetic acid solution and were placed onto a rocker for 10 min to allow the dye to completely dissolve. The dye solution was then placed into 1.5 mL Eppendorf tubes and vortexed for 30 s before 150 μL of 4 mol·L^−1^ NaOH was added, and the pH was adjusted to 4.1. The tubes were then centrifuged at 10 000 r.p.m. for 10 min. Triplicates of 100 μL were transferred to a 96-well plate, and the absorbance was read at 405 nm.

### *In vivo* study of bone defects

Twelve male Fischer rats (Animal Resources Centre) at 6 weeks of age were used in this study. A 1×1×2 mm^3^ defect in the medial condyles of rat femurs was made with a 1-mm diameter trephine burr. Gentamycin (5 mg·kg^−1^, Life Technologies) was administered intramuscularly to avoid infections in the wound. On postoperative days 1, 4, 7 and 28, the animals were killed, and the femoral condyles were harvested and fixed in 4% paraformaldehyde for biological evaluation.

### Tartrate-resistant acid phosphatase staining

The staining solution was prepared with 0.2 mol·L^−1^ sodium acetate and 50 mmol·L^−1^ L-(+)-tartaric acid, and the pH was adjusted to 5. Deparaffinized sections were incubated in the acetate buffer for 20 min at room temperature. To this same acetate buffer, 0.5 mg·mL^−1^ of naphtol AS-MX phosphate and 1.1 mg·mL^−1^ of fast red TR salt were added, and the sections were incubated for 1 h at 37 °C, followed by counterstaining with Mayer's hematoxylin. Tartrate-resistant acid phosphatase-positive (TRAP+) cells appeared bright red. Qualitative and quantitative histological evaluations of osteoclasts were then performed using ImageJ.

### Data analysis

Data are shown as the mean±s.d. for three independent experiments. Statistical differences among the groups were determined by one-way analysis of variance with Bonferroni’s multiple comparison tests. Any *P*-value<0.05 was considered statistically significant.

## Results

### Similar distributions of molecular markers of M1 and M2 cells in cortical bones

**Figure 1:**
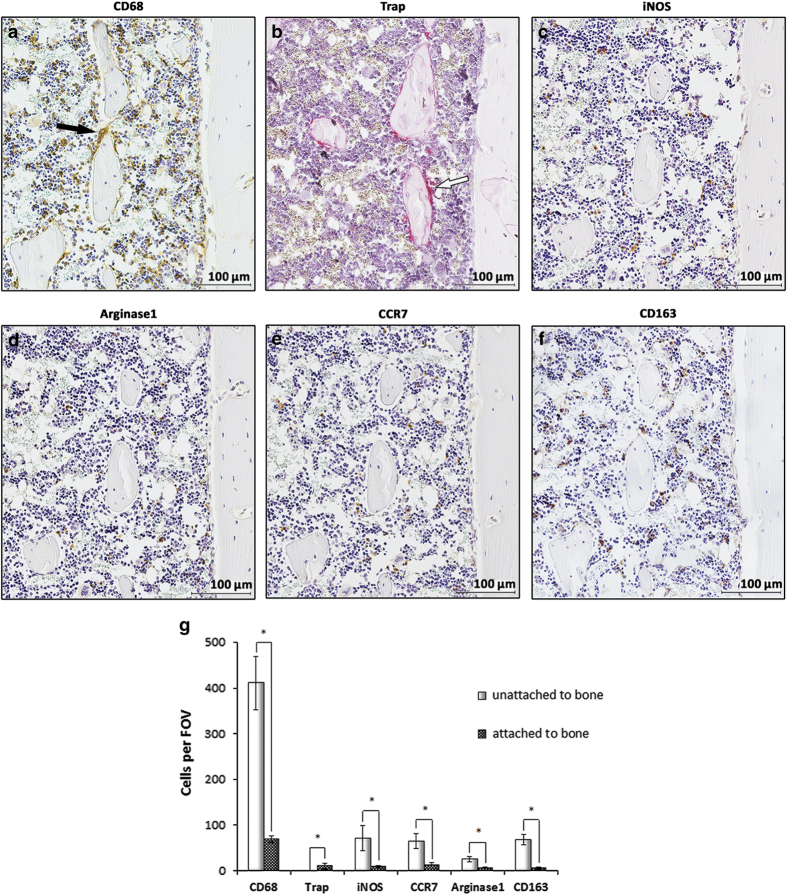
M1 and M2 macrophages showed similar expression patterns in cortical bone. Immunohistochemical staining was performed in sagittal serial sections of medial condyles from 6-week-old rats within the diaphyseal region. Cluster of differentiation 68 (CD68) was selected as a pan marker for the macrophage lineage, including monocytes, macrophages, giant cells, and osteoclasts. Inducible nitric oxide synthase (iNOS) and C-C chemokine receptor type 7 (CCR7) were selected as M1 phenotypic markers, whereas Arginase1 and cluster of differentiation 163 (CD163) were selected as M2 phenotypic markers. Tartrate-resistant acid phosphatase (TRAP) staining was performed as described in Materials and Methods. The distribution of CD68+ cells was divided into two patterns, one of which revealed the cells were mainly in the reticular tissue, and the other showed the cells were attached to bone surfaces to form a canopy-like structure over the osseous tissue. CD68+ (black arrows) and TRAP+ (white arrows) cells were not co-localized in cortical bone (**a**, **b**). Most of the M1 and M2 antibody-labeled cells were regular in shape and were not attached to the bone surfaces (**c**–**f**). The quantification of the cell numbers according to their location revealed that most of the M1 and M2 cells were not attached to the bone surfaces (**g**). Representative images from three independent experiments are shown. FOV, field of view. Data shown as the mean±s.d. (*P<0.05).

The distribution of macrophages and their phenotypes were investigated in the serially sectioned cortical bone of rat femurs as illustrated in Figure 1. CD68 is a marker for the macrophage lineage, including monocytes, macrophages, giant cells, and osteoclasts.^17^ iNOS and CCR7 are markers for M1 macrophages, whereas Arginase1 and CD163 are markers for M2 macrophages.^18,19^ Representative stained sections showed that cells expressing CD68 (black arrows) were distinct from TRAP+ cells (white arrows) within this endosteal bone environment (Figure 1a and b). Cells labeled by the antibodies against iNOS, CCR7, Arginase1, and CD163 shared similar expression patterns with regular cell morphology (Figure 1c–f). Histomorphometric quantification illustrated that no significant difference was found between the numbers of M1 and M2 cells. Most of these cells were not attached to bone surfaces (Figure 1g).

### Most M1 cells are attached to the trabecular bone surface but M2 cells are mainly identified in bone marrow

**Figure 2:**
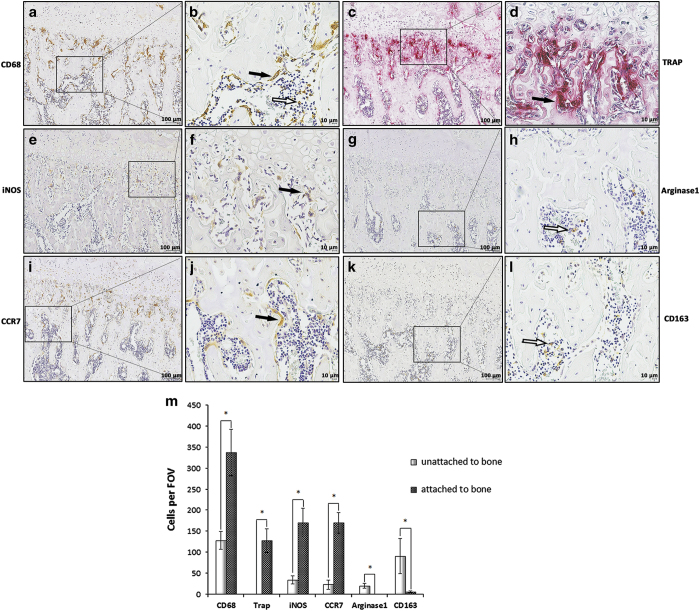
M1 expression pattern changed in trabecular bone. Immunohistochemical staining was performed in sagittal serial sections of medial condyles from 6-week-old rats within the epiphyseal region. The region with the solid line indicates areas of interest to be enlarged in the following image. Representative staining with the anticluster of differentiation 68 (CD68) antibody demonstrated that CD68+ cells were either attached (black arrows) or unattached (white arrows) to the bone surfaces with a variety of sizes and shapes (**a**, **b**). TRAP+ cells showed multinucleated morphology (black arrows) which is similar to the CD68+ cells (**c**, **d**). Inducible nitric oxide synthase-positive (iNOS+) and C-C chemokine receptor type 7-positive (CCR7+) cells (black arrow) were attached to the bone surfaces with an elongated morphology (**e**, **f**, **i**, **j**). Arginase1+ and cluster of differentiation 163-positive (CD163+) cells (white arrow) represented the predominant population in the reticular connective tissue with similar expression patterns as in the cortical bone (**g**, **h**, **k**, **l**). No positive staining was found in isotype controls (data not shown). Most of the M1-labeled cells were found attached to bone surfaces instead of M2-labeled cells (**m**). Representative images from three independent experiments are shown. FOV, field of view. Data shown as the mean±s.d. (*P<0.05).

The distribution of macrophages and their phenotypes were examined in serially sectioned medial condyles of rat femurs, as illustrated in Figure 2. Confined regions with solid lines indicate areas of interest to be enlarged. Cells labeled by the antibody anti-CD68 were found to be attached (black arrows) or unattached (white arrows) to bone surfaces and displayed a large diversity of cell morphology and arrangements (Figure 2a and b). TRAP+ cells showed a multinucleated morphology with bright red color on bone surfaces and comprised a subset of CD68-positive macrophages (Figure 2c and d). M1 cells labeled by the antibodies anti-iNOS and anti-CCR7 were found attached to bone surfaces with elongated cell shapes (Figure 2e, f, i, and j). Conversely, M2 cells labeled by the antibodies anti-Arginase1 and anti-CD163 were primarily located a certain distance away from the growth plate, most of which were found in reticular tissues with regular cell morphology (Figure 2g, h, k, and l). Most of the M1 cells were found attached to bone surfaces, whereas M2 cells were unattached to bone surfaces (Figure 2m).

### iNOS-positive cells were found in areas of increased RANKL expression

**Figure 3:**
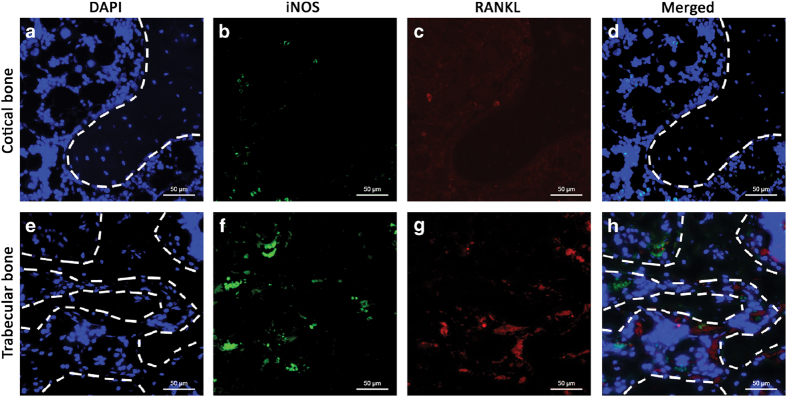
Inducible nitric oxide synthase-positive (iNOS+) cells were found in areas of increased RANKL expression. Samples collected from cortical bone and trabecular bone were stained with anti-iNOS and anti-RANKL antibodies and visualized with Alexa Fluor 488 (green) and 568 (red). 4', 6-diamidino-2-phenylindole (blue) was used to identify the nuclei. The dotted line separates the regions of bone and bone marrow. More iNOS+ cells were found in trabecular bone with the elevated expression level of RANKL compared to cortical bone (**a**–**h**). Representative images from three independent experiments are shown.

Cortical and trabecular bone samples from rat femurs were stained with antibodies against iNOS (green) and RANKL (red) and counterstained with 4', 6-diamidino-2-phenylindole (blue). The dotted line divides the regions of bone and bone marrow. Increased immunofluorescent intensities of iNOS and RANKL were found in trabecular bone compared to cortical bone (Figure 3a–h).

### RANKL caused BMDM phenotypic switching *in vitro*

**Figure 4:**
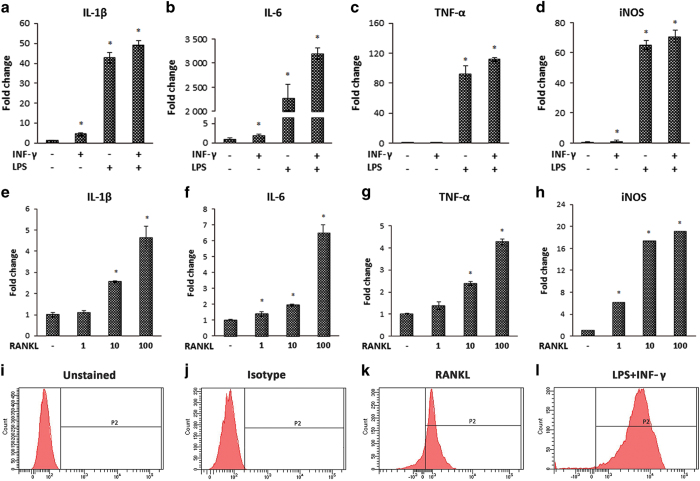
RANKL induced M1 phenotypic switching of macrophages *in vitro*. To evaluate the roles of physiological (RANKL) and pathological [lipopolysaccharide (LPS)] stimuli in macrophage activation, bone marrow-derived macrophages (BMDMs) were induced with LPS, interferon-γ (IFN-γ), LPS+IFN-γ (**a**, **b**, **c**, **d**), and RANKL (**e**, **f**, **g**, **h**), respectively. RANKL was able to elevate the expression of M1-related markers such as interleukin (IL)-1β (**e**), IL-6 (**f**), tumor necrosis factor (TNF)-α (**g**), and inducible nitric oxide synthase (iNOS) (**h**), although not as strongly as LPS+ IFN-γ (**a**–**h**). Representative histograms of FACS show the peak of iNOS expression induced with RANKL shifted to the right (**k**), although showing lower fluorescent intensity compared with those induced with LPS+IFN-γ (**l**). The results are shown as the mean ±s.d. from three independent experiments performed in triplicate (*P<0.05).

To compare the roles of pathological and physiological stimuli in macrophage activation, BMDMs were stimulated with LPS, IFN-γ, LPS+IFN-γ, and RANKL, respectively. The expression of M1 markers such as *IL-1β*, *IL-6*, *TNF-α*, and *iNOS* were elevated when the cells were treated with RANKL. However, these RANKL-induced cells may not acquire the full spectrum of polarization because of the lower levels of pro-inflammatory cytokine expression compared with those induced by LPS+IFN-γ (Figure 4a–h). To further confirm our finding, FACS was used to analyze iNOS expression. Representative histograms showed that the peak in RANKL-stimulated samples shifted to the right, with lower fluorescent intensity compared with those stimulated with LPS+IFN-γ (Figure 4i–l).

### RANKL-CM accelerated MSC osteogenic differentiation *in vitro*

**Figure 5:**
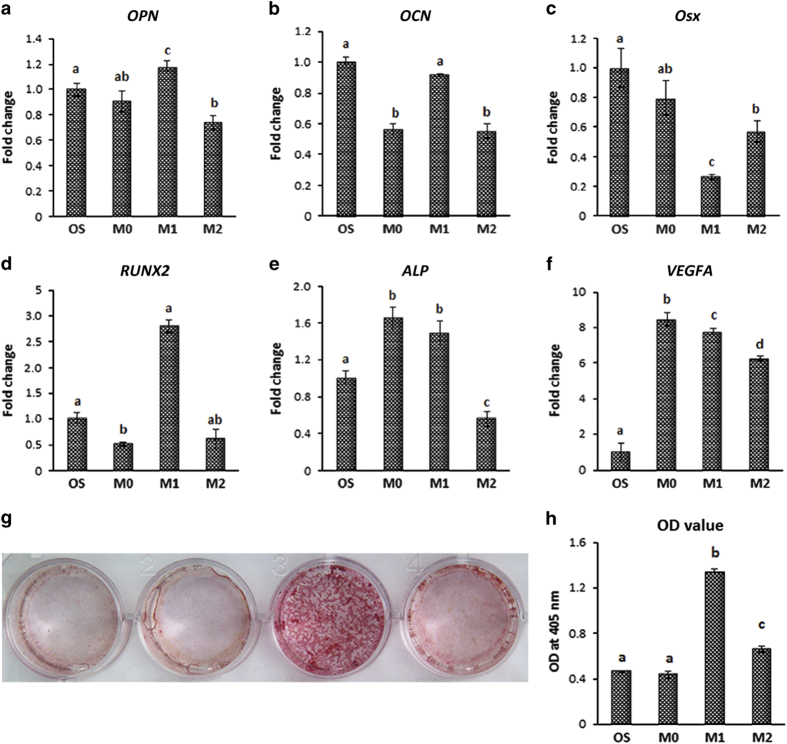
Conditioned medium promoted osteogenic differentiation of MSCs. To examine the hypothesis that macrophage subtypes affect the osteogenic ability of MSCs, macrophages were left untreated (M0) or treated with RANKL (M1) and IL-4 (M2) for 7 days. The expressions of osteogenic markers were assessed by reverse transcription-quantitative polymerase chain reaction (RT-qPCR). The mRNA levels of *OPN*, *RUNX2*, *ALP*, and *VEGF* were enhanced by M1-conditioned medium compared with the controls (**a**–**f**). The samples were stained with Alizarin Red S to visualize mineralization. The intensity of matrix mineralization was significantly increased in the M1-conditioned group (**g**, **h**). The results are shown as the means ±s.d. from three independent experiments performed in triplicate. The means marked with different letters are significantly different (*P<*0.05).

To explore the role of macrophages in osteogenesis, MSCs were cultured with osteogenic differentiation medium supplemented with macrophage (M0)-, RANKL (M1)-, or IL-4 (M2)-CMs for 7 days. The osteogenic gene expression of MSCs was analyzed by RT-PCR. A modest increase was found in the expression of osteogenesis-related genes, such as *OPN*, *RUNX2*, *ALP,* and *VEGF*, in MSCs that had been co-cultured with M1-CM (Figure 5a–f). Alizarin Red S staining was performed to visualize the mineralized nodules. After 7 days of incubation, stronger red staining appeared in the M1-conditioned group, which indicated that MSCs cultured with M1-CM had a greater tendency for calcified nodule formation (Figure 5g and h).

### Osteoclasts participated in bone healing

**Figure 6:**
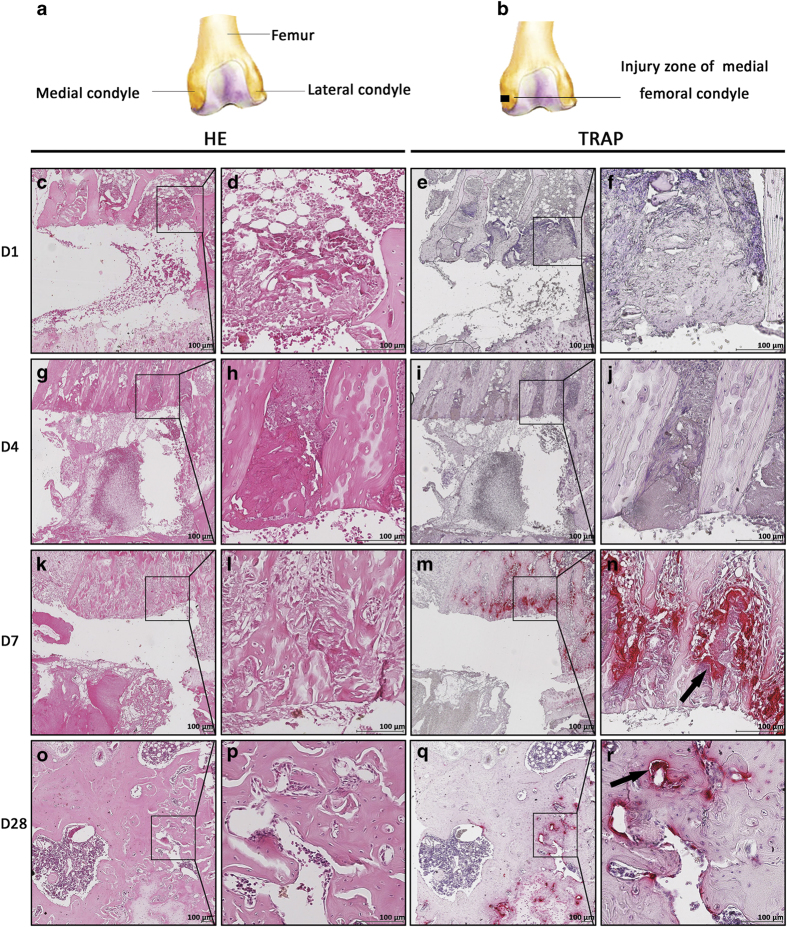
Osteoclasts had a stage-dependent role in bone healing. RANKL signaling regulates osteoclast formation. To understand the involvement of osteoclasts, bone defects were created as illustrated (**a**, **b**). Sections at different time points were stained with hematoxylin and eosin (H&E) and tartrate-resistant acid phosphatase (TRAP). The region surrounded by the solid line indicates areas of interest to be enlarged in the following picture. Representative staining showed that no TRAP+ cells were found on days 1 or 4 post surgery (**c**–**j**). The presence of TRAP+ staining started on postoperative day 7 and returned to normal on day 28 (**k**–**r**; black arrows), which is consistent with the transition time of macrophage transformation into osteoclasts. Representative images from three independent experiments are shown.

A 1×1×2 mm^3^ defect was created near the growth plate in the rat medial condyle (Figure 6a and b). The sections were stained with hematoxylin and eosin and TRAP. No positive staining was found on the first or fourth days of bone healing (Figure 6c–j). The presence of TRAP-positive cells that gathered around the bone defect with bright red color started on postoperative day 7 and returned to normal on day 28 (Figure 6k–r; black arrows), indicating a stage-dependent role of osteoclasts during bone healing.

### M1 cells predominated in early bone healing

**Figure 7:**
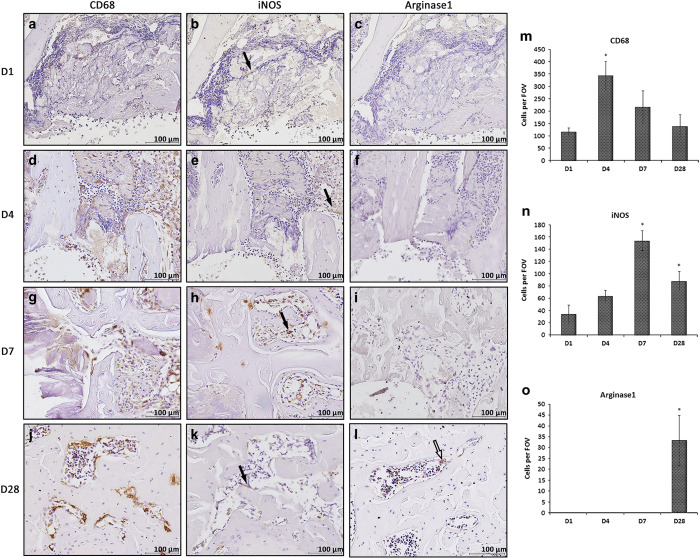
M1 macrophages were actively involved in early bone healing. Antibodies of anti-CD68, anti-inducible nitric oxide synthase (iNOS; black arrows), and anti-Arginase1(white arrows) were applied in the condyle defect model. Cluster of differentiation 68 (CD68)-labeled cells were present on postoperative day 1 and peaked on day 4 post surgery (**a**, **d**, **g**, **j**, **m**). iNOS-labeled cells were found on postoperative day 1 and peaked on day 7 post surgery (**b**, **e**, **h**, **k,** and **n**); Arginase1-labeled macrophages only infiltrated the healed region on day 28 (**c**, **f**, **i**, **l**, **o**). Representative images from three independent experiments are shown. FOV, field per view. Data shown as the mean±s.d. (*P<0.05).

Immunohistochemical studies for the 1×1×2 mm^3^ defects revealed that CD68+ and iNOS+ cells were present around the bone defect and persisted throughout the healing process. Cells that stained highly positive for CD68 were more predominant on postoperative day 4 compared with other time points (Figure 7a, d, g, and j). An increase in the mobilization of iNOS+ cells (black arrows) to the defect site was found from 4 to 7 days postoperatively with irregular cell morphology. Specifically, the number of M1 cells increased on day 4, peaked on day 7, and decreased thereafter (Figure 7b, e, h, and k), whereas Arginase1-positive cells (white arrows) only appeared on day 28 with a regular, rhombic cell morphology (Figure 7c, f, i, and l). The number of CD68-, iNOS-, and Arginase1-positive cells peaked on postoperative days 4, 7, and 28, respectively (Figure 7m–o), suggesting the potential transition time for different macrophage subtypes during bone healing with potentially distinct functions.

### Schematic illustration of the potential network of bone marrow-derived tissue macrophages

**Figure 8:**
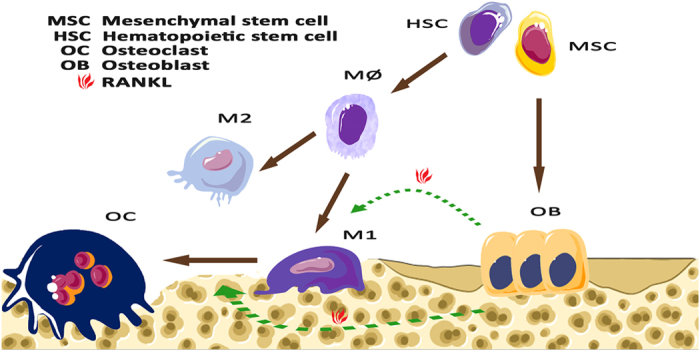
Schematic illustration of the potential phenotypic switching of macrophages. RANKL induces the polarization of macrophages into the M1 phenotype that is present in normal long bones. Conversely, interleukin (IL)-4, IL-10, IL-13, and other glucocorticoids may promote the resolution of inflammation by skewing macrophages toward the M2 phenotype. M1 macrophages may be in a transient state that is similar to the pre-osteoclast state and finally turn into osteoclasts through the persistent induction of RANKL in the physiological environment.

The schematic diagram shows the interconnected network among macrophages, bone cells, and their progenitors (Figure 8). RANKL induces phenotypic switching in macrophages from the initial state to M1-like cells, whereas IL-4, IL-10, IL-13, and other cytokines secreted by T helper cells are involved in M2 activation. Macrophages may undergo the M1 state in sustained RANKL induction and then differentiate into osteoclasts.

## Discussion

In addition to being immune sentinels on the frontline of immune defense, macrophages as a heterogeneous population are tissue-specific and are accurately positioned and transcriptionally programmed for encounters with environmental challenges.^20^ An analysis of the immune context, such as the location and functional orientation of macrophages and how they are integrated with local microenvironment, can provide important information and facilitate predictions of the macrophage response to various treatments.^21^ Tissue macrophages were reported to promote intramembranous bone healing in a mouse tibial injury model.^22^ Studies of macrophage subtypes show that during bone repair, inflammatory macrophages and resident macrophages coexist within the injury site.^23^ M2 macrophages were reportedly prevalent during the ossification phase in the process of fracture healing. Therefore, researchers think that the enhancement of the M2 phenotype in macrophages is an approach to promote bone healing.^24^ Our experiment provides evidence that M1 and M2 tissue macrophages coexist in rat long bones. Macrophages that highly expressed M1 markers, however, consistently failed to express M2 markers. M1 and M2 macrophages shared similar expression patterns in cortical bone, but M1 macrophages showed a strikingly different morphology and distribution in trabecular bone near the growth plates. Bone remodeling is more active in the epiphyseal growth plate by a finely balanced cycle of cartilage growth, matrix formation, and cartilage calcification that enables long bones to increase in diameter and change shape.^25^ Combined with the anatomic location of M2 cells, these observations reveal that M1 cells may be more actively involved in the anabolic modeling of long bones. Interestingly, a number of M1 cells demonstrated an irregularly elongated shape distinct from that of normal macrophages and osteoclasts, which suggests that these elongated cells may exist in a unique state with potentially distinct functions.

The term “classical activation” of macrophages was first introduced in an infection context to describe the antigen-dependent microbicidal activity of macrophages toward bacillus Calmette-Guerin.^26^ M1 activation can be achieved *in vitro* when IFN-γ-primed cells encounter microbial products such as LPS by interacting with the extracellular receptors, resulting in a series of signaling cascade activations and the secretion of pro-inflammatory cytokines including IL-1β, IL-6, and TNF-*α*, and pro-inflammatory mediators such as prostaglandin E2 and NO. The factors secreted after IFN-γ and LPS stimulation cooperatively regulate the transcription of their target genes, leading to the full priming of macrophages.^27–29^ However, unlike the M2-dependent cytokines IL-4, IL-10, and IL-13, which are secreted by T lymphocytes and can be present under normal physiological conditions, LPS is a major constituent of Gram-negative bacteria and is mainly present in pathological states.^30^ Therefore, it is unreasonable to simply explain the presence of M1 cells through the stimulation of LPS. However, in physiological situations *in vivo*, cells may be slightly activated as a selective advantage and may not attain the full spectrum of functional capabilities.

The immunofluorescent results show that the number of iNOS-positive cells increased with the elevated expression level of RANKL, suggesting a potential relationship between M1 cells and RANKL. The *in vitro* data further confirm that BMDMs experience a similar M1 alteration during osteoclastic differentiation. Treatment with RANKL increased the expression of pro-inflammatory cytokines and iNOS in macrophages, but not as strongly as LPS+IFN-γ. This result indicates that M1 activation can be achieved by RANKL in the absence of LPS and IFN-γ. Osteoclastic precursors are known to produce large amounts of pro-inflammatory cytokines and chemokines during differentiation induced by RANKL.^31,32^ These pro-inflammatory cytokines and chemokines, such as IL-1β, IL-6, and TNF-*α*, are markers for M1 macrophages. Therefore, M1 macrophages may be associated with the presence of RANKL while differentiating into osteoclasts. These RANKL-induced macrophages are semi-activated M1-like cells that may have different properties compared with the fully activated ones induced by LPS+IFN-γ.

Because M1 macrophages and bone cells are in close physical contact in the trabecular bone, we sought to determine the impact of macrophage subtypes on osteogenesis *in vitro*. The balance between immune and skeletal systems can be affected by the inflammatory state of the local microenvironment. In this regard, differential subtypes of macrophages are postulated to have an important role in regulating the osteogenic differentiation of MSCs.^33^ Our results show that co-culture with RANKL-CM elevated the osteogenic ability of MSCs, as indicated by the increases in bone-related gene expression and matrix mineralization, which suggest that the transient inflammatory phase M1-like cells brought in at the early stage of osteogenesis may be crucial for enhanced bone formation.

Osteoclasts are terminal cells that specialize in bone resorption, which is irreplaceable for the continual physiological process of bone remodeling.^34^ More importantly, osteoclasts are committed to the induction of RANKL through the receptor activator of nuclear factor *κ*B (RANK),^12^ which was found to be associated with M1 macrophages in our present study. To identify the roles of bone-specific osteoclasts and RANKL-activated M1 macrophages in natural bone healing, a bone defect model was established. The osteoclastic activity surrounding bone defects was not observed at the very beginning of bone healing. The number of osteoclasts increased markedly on the seventh day post surgery and decreased at the late-stage post injury, thus implying a certain correlation between osteoclastic activity and the emergence of bone formation.

Studies have shown that macrophage subtypes are required for tissue repair and bone remodeling and that the release of pro-inflammatory cytokines by macrophages has a vital role in early fracture repair.^23,35^ Our study suggests that CD68+ cells present in the defect first (maximum on day 4) and may then be polarized to M1 macrophages or become osteoclasts, resulting in the M1 and osteoclasts peaks on day 7. The number of macrophages and M1 cells peaked early and returned to normal accompanied by the appearance of M2 cells. Other studies suggest that some M2 macrophages present at wound-healing sites were originally M1 macrophages *in vivo* and that the modulation of macrophage phenotypes from M0 to M2 and M1 to M2 induced by IL-4 can be achieved *in vitro*.^33,36^ All available lines of evidence show that a proper balance of an inflammatory state (M1) followed by an anti-inflammatory state (M2) is critical for normal bone healing. Indeed, there were similar morphological and phenotypic shifts of macrophages during bone healing in our study. As macrophages can undergo dynamic transitions between different functional states, it is possible that a balance between M1/M2 phenotypes underlies these states to maintain bone homeostasis. Although M2 cells can be generated by T lymphocyte-secreted cytokines, the precise origin and capability of M2 cells still require further investigation.

Altogether, our study reveals a variable and multifaceted profile of tissue macrophages, which goes beyond the well-characterized immune cells. We have demonstrated that physiological stimuli can induce the M1 subtype of macrophages. Compared with pathologically induced macrophages, they display distinct properties and may perform specialized, tissue-specific functions. These findings have substantiated the importance of tissue macrophages, particularly the M1 macrophages, and have provided new insights into inflammation control and potential intervention during bone healing.

## Figures and Tables

**Table 1:** Primer sequences

Genes	Primer sequence (5′–3′)
*IL-1β*	TGGAGAGTGTGGATCCCAAG
	GGTGCTGATGTACCAGTTGG
*IL-6*	ATAGTCCTTCCTACCCCAATTTCC
	GATGAATTGGATGGTCTTGGTCC
*TNF-α*	CTGAACTTCGGGGTGATCGG
	GGCTTGTCACTCGAATTTTGAGA
*iNOS*	TGGTGAAGGGACTGAGCTGT
	CTGAGAACAGCACAAGGGGT
*OPN*	CAATGAAAGCCATGACCACATGG
	CTCATCTGCGGCATCAGGATACTG
*OCN*	ACCTAGCAGACACCATGAGGAC
	RGGGGACTGAGGCTCCAAG
*Osx*	AGCGACCACTTGAGCAAACAT
	GCGGCTGATTGGCTTCTTCT
*RUNX2*	AGGGACTATGGCGTCAAACA
	GGCTCACGTCGCTCATCTT
*ALP*	GGACAGGACACACACACACA
	CAAACAGGAGAGCCACTTCA
*VEGFA*	AACGATGAAGCCCTGGAGTG
	GACAAACAAATGCTTTCTCCG
*β-Actin*	5 CATACCCAAGAAGGAAGGCTGG
	GCTATGTTGCTCTAGACTTCGAGC
